# Supplementary figures and images for: Expression of pyrethroid metabolizing P450 enzymes characterizes highly resistant Anopheles vector species targeted by successful deployment of PBO-treated bednets in Tanzania
Source: PLoS One. 2022 Jan 24;17(1):e0249440. doi: 10.1371/journal.pone.0249440 (PMC8786186; doi:10.1371/journal.pone.0249440)

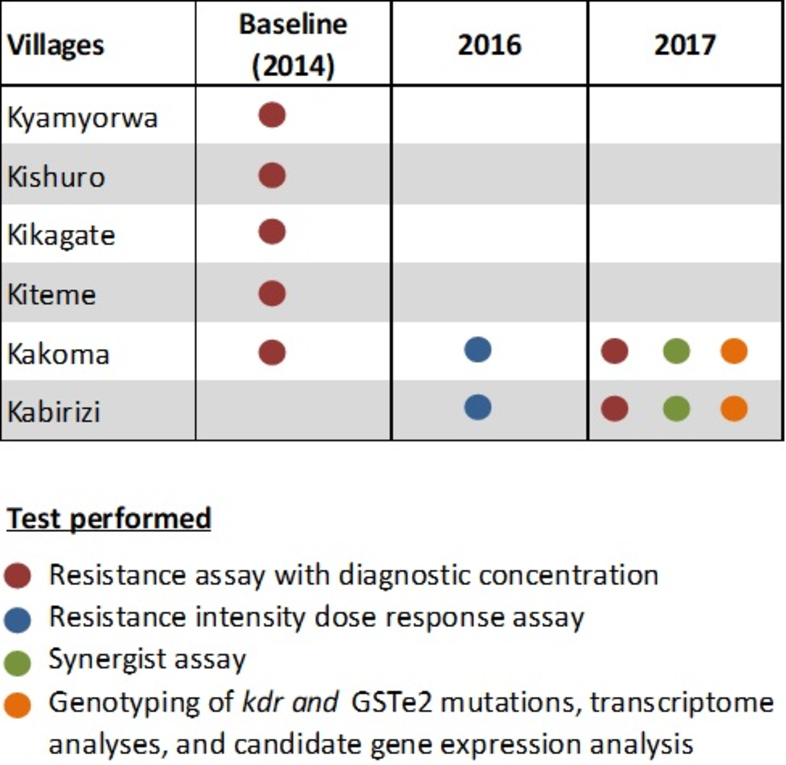

Supplement: S1 Fig — (TIF) [file pone.0249440.s001.tif]

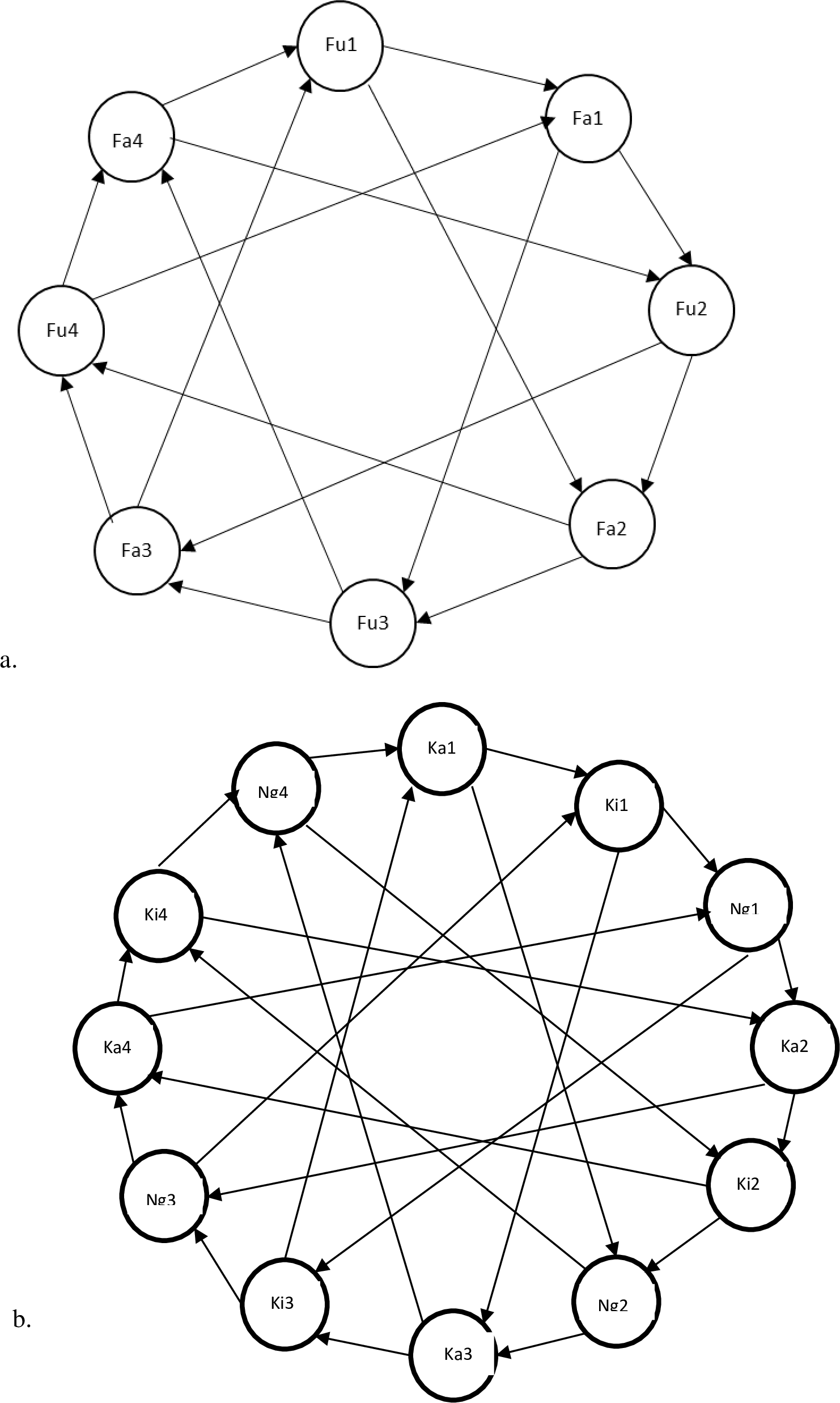

Supplement: S2 Fig — (TIF) [file pone.0249440.s002.tif]
