# Supplementary material for: Expression of pyrethroid metabolizing P450 enzymes characterizes highly resistant Anopheles vector species targeted by successful deployment of PBO-treated bednets in Tanzania
Source: PLoS One. 2022 Jan 24;17(1):e0249440. doi: 10.1371/journal.pone.0249440 (PMC8786186; doi:10.1371/journal.pone.0249440)
Supplement: S1 Table — (DOC) [file pone.0249440.s003.doc]

**S1 Table**

| Gene | Primer sequence | Reference |
| --- | --- | --- |
| CYP6N1 | F: 5’ GAAGCATTTCCGTTTTACGC - 3’ | This paper |
|  | R: 5’- CGGTGGCTTTATAGCTCGTT - 3’ |  |
| CYP6M7 | F: 5’-ACGACGGTACGCTAACGACT-3’ | This paper |
|  | R: 5’-TAACGCCAGCTCATACAACG -3’ |  |
| CYP6M1 | F: 5’- CGACCATCGGGTGAAGAACA-3’ | This paper |
|  | R: 5’- ATGTGAGCGATTCTTCCGGG-3’ |  |
| CYP6Z1 | F: 5’- AACGCGCTGAAGTATCTGGA -3’ | This paper |
|  | R: 5’- TGGTGTCCCCTTGCGAATGA -3’ |  |
| S7 | F: 5’- GTGTTCGGTTCCAAGGTGAT -3’ | This paper |
|  | R: 5’- TCCGAGTTCATTTCCAGCTC -3’ |  |
| Actin | F: 5’- TTAAACCCAAAAGCCAATCG 3’ | This paper |
|  | R: 5’- ACCGGATGCATACAGTGACA -3’ |  |
