# Supplementary material for: Expression of pyrethroid metabolizing P450 enzymes characterizes highly resistant Anopheles vector species targeted by successful deployment of PBO-treated bednets in Tanzania
Source: PLoS One. 2022 Jan 24;17(1):e0249440. doi: 10.1371/journal.pone.0249440 (PMC8786186; doi:10.1371/journal.pone.0249440)
Supplement: S2 Table — (DOC) [file pone.0249440.s004.doc]

**S2 Table**

| Gene | Primer sequence | Reference |
| --- | --- | --- |
| CYP6P3 | F: 5’-GTGATTGACGAAACCCTTCGGAAGT-3’ | Witzig *et al.,*2013 |
|  | R: 5’-GCACCAGTGTTCGCTTCGGGA-3’ |  |
| CYP6M2 | F: 5’-TACGATGACAACAAGGGCAAG- 3’ | Witzig*et al.,*2013 |
|  | R: 5’- GCGATCGTGGAAGTACTGG-3’ |  |
| CYP6P4F | F: 5’- GTCTGCGGGAGGAAATCGAG - 3’ | This paper |
|  | R: 5’- TACTTGCGCAGGGTTTCATTG -3’ |  |
| CYP9K1. | F: 5’- GAAGCGGTCGGTTGACTGGA-3’ | This paper |
|  | R: 5’- TCGTGCGCCATAAATGCAGA -3’ |  |
| CYP6Z3 | F: 5’- CCACGCAATTGCATTGGTCTT - 3’ | This paper |
|  | R: 5’- CAACTACAGTAGCAACAGCAAAT -3’ |  |
| CYP9J5 | F: 5’- AAACCGATACCCTCGTTGGC - 3’ | This paper |
|  | R: 5’- CCGAAAACTTTCACCAGCGG -3’ |  |
| GSTe2 | F: 5’- TGTGAAGCTAAACCCGCAAC - 3’ | This paper |
|  | R: 5’- CTTGACGGGGTCTTTCGGAT -3’ |  |
| VATPase | F: 5’-AACCAGGAAGCCGAAGCTAA - 3’ | This paper |
|  | R: 5’- TGGTACGTGCCTTAGAACGG -3’ |  |
| CYP6AA1 | F: 5’- CAACTCCACGACGGCAAGAT - 3’ | This paper |
|  | R: 5’- CGGATACATACGAAGTGTCTCATT-3’ |  |
| CYP6M1 | F: 5’- GTGCTCGCCAAGCATAATGG - 3’ | This paper |
|  | R: 5’- ACTTGCGTAGGGATTCTTTCA -3’ |  |
| S7 | F: 5’- AGAACCAGCAGACCACCATC-3’ | Jones et *al.,*2013 |
|  | R: 5’- GCTGCAAACTTCGGCTATTC-3’ |  |
| EF | F: 5’- AGCAGCTGTTCAGCAAAACG -3’ | This paper |
|  | R: 5’- TCTCCCGCACAGTGAAAGAC -3’ |  |
|  |  |  |
