# Supplementary material for: Expression of pyrethroid metabolizing P450 enzymes characterizes highly resistant Anopheles vector species targeted by successful deployment of PBO-treated bednets in Tanzania
Source: PLoS One. 2022 Jan 24;17(1):e0249440. doi: 10.1371/journal.pone.0249440 (PMC8786186; doi:10.1371/journal.pone.0249440)
Supplement: S4 Table — (DOC) [file pone.0249440.s006.doc]

**S4 Table Characteristics of *Anopheles funestus* genes chosen for further investigation by quantitative PCR including summarised results from the microarrays (averages across replicate probes on the array)**

| **Gene** | **Function**  **(Group)** | **Accession number** | **Location**  **(Chromosome)** | **Mean Fold change** |  | **Mean corrected p value** |  |
| --- | --- | --- | --- | --- | --- | --- | --- |
| CYP6M1 | Cytochrome P450 | AFUN010921 | 2 | 15.7 |  | 8.7 x10^-8^ |  |
| CYP6M7 | Cytochrome P450 | AFUN015795 | 2 | 3.7 |  | 5.8 x10^-8^ |  |
| CYP6N1 | Cytochrome P450 | AFUN010918 | 2 | 21.9 |  | 1.4 x10^-8^ |  |
| CYP6Z1 | Cytochrome P450 | AFUN015919 | 2 | 4.8 |  | 7.4 x10^-8^ |  |
