# Supplementary material for: Expression of pyrethroid metabolizing P450 enzymes characterizes highly resistant Anopheles vector species targeted by successful deployment of PBO-treated bednets in Tanzania
Source: PLoS One. 2022 Jan 24;17(1):e0249440. doi: 10.1371/journal.pone.0249440 (PMC8786186; doi:10.1371/journal.pone.0249440)
Supplement: S7 Table — (DOC) [file pone.0249440.s009.doc]

**S7 Table. Characteristics of *Anopheles gambiae* genes chosen for further investigation by quantitative PCR including summarised results from the microarrays (averages across replicate probes on the array and both experiments)**

| **Gene** | **Function**  **(Group)** | **Accession number** | **Location**  **(Chromosome)** | **Mean Fold**  **change** | **Mean corrected p value** |
| --- | --- | --- | --- | --- | --- |
| CYP6P3 | Cytochrome P450 | AGAP002865 | 2R | 6.8 | 7.7 x10^-8^ |
| CYP6P4 | Cytochrome P450 | AGAP002867 | 2R | | 4.2 | | --- | | | 1.11 x10^-8^ |
| CYP6AA1 | Cytochrome P450 | AGAP002862 | 2R | | 4.8 | | --- | | | 1.3E x10^-7^ |
| CYP9K1 | Cytochrome P450 | AGAP000818 | X | | 9.4 | | --- | | | 4.2 x10^-9^ |
| CYP6Z3 | Cytochrome P450 | AGAP008217 | 3R | | 4.6 | | --- | | | 3.1 x10^-6^ |
| CYP6M2 | Cytochrome P450 | AGAP008212 | 3R | | 10.7 | | --- | | | 2.4 x10^-6^ |
| CYP9J5 | Cytochrome P450 | AGAP012296 | 3L | | -10.2 | | --- | | | 1.2 x10^-8^ |
| GSTe2 | Glutathione-S-transferase | AGAP009194 | 3R | | -10.1 | | --- | | | 1.2 x10^-7^ |
| CYP6M1 | Cytochrome P450 | AGAP008209 | 2L | | -3.2 | | --- | | | 4.7 x10^-2^ |
