# Supplementary material for: Expression of pyrethroid metabolizing P450 enzymes characterizes highly resistant Anopheles vector species targeted by successful deployment of PBO-treated bednets in Tanzania
Source: PLoS One. 2022 Jan 24;17(1):e0249440. doi: 10.1371/journal.pone.0249440 (PMC8786186; doi:10.1371/journal.pone.0249440)
Supplement: Attachment — Submitted filename: Rebuttal letter_PLOS ONE.pdf [file pone.0249440.s011.pdf]

Rebuttal letter in response to reviewers' and editorial comments on the manuscript titled:
Insecticide resistance characteristic of Anopheles vector species successfully controlled by deployment of pyrethroid and PBO long lasting insecticidal treated nets in Tanzania

Dear Editor,

We have revised our paper in line with the reviewers' and editorial comments. We hope the changes and responses meet the expectation of *PLOS ONE* and the manuscript is now suitable for publication.

Yours sincerely,

Dr. Johnson Matowo (PhD)

Responses to editorial comments

1) Please review your reference list to ensure that it is complete and correct. If you have cited papers that have been retracted, please include the rationale for doing so in the manuscript text, or remove these references and replace them with relevant current references. Any changes to the reference list should be mentioned in the rebuttal letter that accompanies your revised manuscript. If you need to cite a retracted article, indicate the article's retracted status in the References list and also include a citation and full reference for the retraction notice.

Response: *We have checked the reference list and it is complete and correct*

2) Please ensure that your manuscript meets PLOS ONE's style requirements, including those for file naming. The PLOS ONE style templates can be found at

https://journals.plos.org/plosone/s/file?id=wjVg/PLOSOne_formatting_sample_main_body.pdf
and

https://protect-eu.mimecast.com/s/YVgEC7XRKiz0Ew9iqNgnM?domain=journals.plos.org

Response:

We have carefully looked at the style manuscript and hope we are meeting PLOS ONE style requirements.

3) Please clarify whether any permits were necessary/obtained to collect mosquitoes from the specified areas.

Response: *Written consent was obtained from household heads before collecting mosquitoes inside the house. Ethical approval was obtained from the Ethical Review Committee of the Tanzanian Medical Research Coordinating Committee (registration number NIMR/HQ/R.8a/Vol IX/1803) and the LSHTM as well as Kilimanjaro Christian Medical University College. A Material transfer agreement was signed between the project PI at Kilimanjaro Christian*

Medical University College and Liverpool School of Tropical Medicine and approved by the Tanzanian ERC. The transfer of samples to LSTM took place prior to the Republic of Tanzania's signature of the Nagoya protocol (19/4/2018).

4) Please provide a citation for the clinical trail that mosquitoes were collected during.

Response: *Citation 16 refer to the clinical trial and is included in the manuscript"Protopopoff N, Mosha JF, Lukole E, Charlwood JD, Wright A, Mwalimu CD, et al. Effectiveness of a long-lasting piperonyl butoxide-treated insecticidal net and indoor residual spray interventions, separately and together, against malaria transmitted by pyrethroid-resistant mosquitoes: a cluster, randomised controlled, two-by-two factorial design trial. Lancet. 2018;391(10130):1577-88."*

5) We note that you have stated that you will provide repository information for your data at acceptance. Should your manuscript be accepted for publication, we will hold it until you provide the relevant accession numbers or DOIs necessary to access your data. If you wish to make changes to your Data Availability statement, please describe these changes in your cover letter and we will update your Data Availability statement to reflect the information you provide.

Response:
We would like to change the data availability statement to "The microarray data generated are deposited in Array Express with accession numbers E-MTAB-10579 and E-MTAB-10580. Microarray analysis results and quantitative PCR data and results are provided within the supplementary materials.

6) Please amend either the title on the online submission form (via Edit Submission) or the title in the manuscript so that they are identical.

Response: *Done*

7) Please upload a copy of Supporting Information Figure S1 which you refer to in your text on page 25.

Response: *Done*

<u>Responses to Reviewer 1</u>

The research article by Matowo et al is a comprehensive study regarding the insecticide status and related mechanisms operating in a targeted area in Tanzania, where a permethrin/PBO co treated LLINs trial occured. It provides important and operationally relevant information regarding insecticide resistance status which are applicable to relevant trials and field settings. The manuscript is well written and well organized and I only have the following suggestions to make:

1. The relevance OPs/ carbamates bioassays performed in this study (otherwise focusing on pyrethroid resistance) could be more elaborate to introduce the reader to the concept of cross

resistance. Since no molecular diagnostics for OPs/carabamates have been performed, target site mechanisms should be discussed as a possible mechanism. The authors mention that no ace mutations had been detected in the area based on data from 2013, but this could have changed in 5-10 years' time.

Response:

As suggested by the reviewer we have reviewed the discussion and amended to the following:

Interestingly, carbamate resistance increased significantly in An. gambiae between 2014 and 2017, despite cessation of carbamate use for control prior to the baseline collections. The presence of acetylcholinesterase (Ace1) target site mutation has been involved in resistance to organophosphates and/or carbamates in Anopheles populations [49]. Bendiocarb resistance was already observed in the study site in 2011 while Ace1 mutations was not detected at this time in local An. gambiae [19]. In the present study, Ace 1 was not investigated and could have contributed to the change in bendiocarb resistance. Pyrethroid-driven over expression of P450s may cause or perhaps combine with Ace-1 mutations to produce bendiocarb resistance, with the primary cross-resistance candidate genes CYP6M2 and CYP6P3 in An. gambiae's sister species An. coluzzii [49], and CYP6Z1 in An. funestus [46] all significantly overexpressed in Muleba populations.

Please note that while making the above revision we noticed that Cyp6Z1 in Figure 4b and S5 Table was erroneously labelled as Cyp6Z3, along with two places in the text. Each instance has been corrected.

2. PBO experiments show clear metabolic resistance, but this could be, as in many other cases, a synergistic phenomenon operating in parallel with other resistance mechanisms (e.g. target site mutations like N1575Y and cuticle resistance)

Response:

We agree and make no attempt to discount this possibility in the manuscript.

3. In some instances, it is difficult to distinguish to which mosquito species the authors refer to (An. gambiae ss, An. coluzzii, or hybrid forms).

Response:

Only An. gambiae s.s. and An. arabiensis are found in Tanzania, An. coluzzi was only used as references strain for microarray and qPCR. We have tried to make that clearer in the text.

4. It would be relevant to discuss why the newly defined CYP6P9a/9b SNPs associated with resistance in *An. funestus* were not assessed (i.e., no CYP6P9a over expression detected)
Response:
Although not necessarily causal, these SNPs are linked to an over expression phenotype: Cyp6P9a and 9b were not over expressed in our study population and therefore the SNPs were

not assessed: these results were discussed in the previous version of the manuscript as shown in the following paragraph below:

"The most significantly over expressed genes in An. funestus were dominated by CYP6 subfamily P450s, most notably CYP6N1, CYP6M7, CYP6M1 and CYP6Z1, all of which have been previously associated with pyrethroid resistance [46, 47]. The two best-known pyrethroid-associated P450s in An. funestus, CYP6P9a and CYP6P9b [48] were, however, not over expressed in Muleba, which thus appears to be a population in which CYP6M7 acts in their stead and which metabolizes pyrethroids with equally high efficiency [14]. The gene expression data for the two important Muleba malaria vectors thus provides strong evidence to validate the third WHO criterion, for deployment. "

5. The continued dependence on pyrethroid-treated LLINs is very relevant and could be discussed in more details (logistics: limited supplies of co-PBO treated nets, high cost, distribution issues, other factors?)

Response:

We agree with the reviewer, and we had included some of this is the Introduction already. There are several issues related to the PBO-LLIN that have not been mentioned in this paper as PBO residual efficacy etc. We did not want to go in too much detail as this was not the aim of the paper.

6. A supplementary Table with the list of primers used in this study would be useful

Response: *S1 and S2 tables have been added with the primers used.*

Responses to Reviewer 2

The paper entitled "Insecticide resistance characteristic of *Anopheles* vector species successfully controlled by deployment of pyrethroid and PBO long lasting insecticidal treated nets in Tanzania" is an interesting piece of work from experts in the field of insecticide resistance and vector control. Cluster randomized trial demonstrated that permethrin LLINs co-treated with the mixed function oxidase inhibitor (PBO) was more efficient than the net treated only with pyrethroid. Bioassay data and molecular analysis undertaken in this study to determine the underlying mechanism of pyrethroid resistance in Anopheles gambiae and An. funestus. Both data were in agreement with LLINs efficacy data. The manuscript well written and the data nicely presented and support the final conclusion. I have no hesitation to recommend this work in its form for publication in PLOS ONE.

Response: *Nothing was raised (to respond to)*

We thank the reviewers for their positive comments
