# Supplementary material for: Expression of pyrethroid metabolizing P450 enzymes characterizes highly resistant Anopheles vector species targeted by successful deployment of PBO-treated bednets in Tanzania
Source: PLoS One. 2022 Jan 24;17(1):e0249440. doi: 10.1371/journal.pone.0249440 (PMC8786186; doi:10.1371/journal.pone.0249440)
Supplement: Attachment — Submitted filename: Response to Reviewers.pdf [file pone.0249440.s012.pdf]

Dear Editor,

We have revised our paper in line with the reviewers' and editorial comments. We hope the changes and responses meet the expectation of Plos One and the manuscript is now suitable for publication.

Yours sincerely,

Dr. Johnson Matowo (PhD)

**Response to academic editor and reviewer(s)**

**a) Response to academic editor**

- ➢ The references list has been reviewed. Minor changes have been done for references no.28,29 and 30 (Previous ref.30 moved to 28; Previous ref.28 moved to 29; Previous ref.29 moved to 30). The list is complete and correct.
- ➢ No retracted papers have been cited
- ➢ The title of the manuscript has been changed, see the tracked changes

**b) Response to reviewer(s)**

i. All comments that were raised in a previous round of review by Reviewer #1 & #2 had been addressed

ii. The following are responses to Reviewer #3

**Reviewer #3:**

Matowo et al. examined the incidence, extent, and management of pyrethroid resistance in Anopheles mosquitoes during 2014-2016 in five villages of the Muleba district in North Tanzania.

As pyrethrioid resistance to permethrin treated insecticidal nets has reduced the efficacy of this approach, augmentation using piperonyl butoxide, a synergist that blocks pyrethroid metabolizing enzymatic activity, was investigated. Four villages were selected to receive 1 of 4 treatments: 1) long-lasting insecticide (permethrin) treated netting (LLIN), 2) LLIN + piperonyl butoxide (Py-PBO), 3) LLIN + and indoor residual spray (IRS) using an organophosphate product Actellic, 4) LLIN + Py PBO + IRS. They then conducted sampling to determine if the nets differentially affected mosquito populations in each village. Using flies from the collection the authors conducted a series of tests: a) a resistance assay using single diagnostic concentrations of permethrin, lambda-cyhalothrin, bendiocarb, and pirimiphos-methyl; b) a synergist bioassay aimed at determining how pre-exposure to piperonyl butoxide affected permethrin resistance values, c) a resistance intensity dose response assay aimed at establishing

resistance curves for two mosquito species from two villages both treated with the permethrin netting LLIN treatment alone, Kakoma and Kabirizi. They also conducted molecular and genetic analyses of wild collected An. gambiae and An. Funestus to determine d) genotyping for kdr and GSTe2 mutations; e) transcriptome analysis of pyrethroid resistant mosquitoes; and f) candidate gene expression analysis. Generally, the manuscript is well written and easy to read.

The introduction fully introduces the concepts needed to understand the goals of the project and the methods are well written as far as what was provided. However, there are a number of problems with the manuscript that make evaluation of the claims difficult.

Most importantly, while the focus of the paper is the efficacy of different netting treatments, the data on trapping efficacy is entirely missing. Even after review, it is unclear to me how the authors planned to assess treatment efficacy and there no data provided to show that the netting was effective at management mosquito populations at all. Rather, the authors devote the results to lab assessments of resistance values using flies caught from these villages. However, even with those data, sampling dates are not provided, and statistical comparisons between groups for the data that are presented are absent. Indeed, while there is a suppression of susceptibility to pyrethroids, in comparison to other chemical classes, between sites, the resistance values appear similar and without statistical comparison, there is no way to determine whether differential treatments affected resistance. In fact, the resistance of flies from the single LLIN treatment at Kakoma appeared similar to those that did include the PBO treatment at Kishuro and Kiteme.

i. The authors could either, A) provide those missing data, if they are available and introduce new analyses, or B) the authors may want to change the wording of their claims and make their manuscript more focused on their explicit goals.

**Response**

The paper did not claim to provide efficacy data, which have been published elsewhere by our team (Protopopoff N, et al. Effectiveness of a long-lasting piperonyl butoxide-treated insecticidal net and indoor residual spray interventions, separately and together, against malaria transmitted by pyrethroid-resistant mosquitoes: a cluster, randomised controlled, two-by-two factorial design trial. Lancet. 2018;391(10130):1577-88.). In the present paper we present a secondary objective of the project which was to provide information on resistance intensity and mechanisms in the local *Anopheles* population that could help to explain the primary results in the RCT.

We think that the objective of the study is clearly stated at the end of the Introduction:
'The present study, was aimed at characterizing molecular and metabolic resistance mechanisms present in intensely pyrethroid resistant populations of An. gambiae and An. funestus, and sought to describe the phenotypic and genetic insecticide resistance profiles of malaria vectors in Muleba.'

We have opted to change the wording of our claims; the following is the proposed title:

"Expression of pyrethroid metabolizing P450 enzymes characterizes highly resistant Anopheles vector species targeted by successful deployment of PBO-treated bednets in Tanzania"

Please see my more detailed line comments below:

ii. Ln 68: Generally, readers struggle to keep multiple acronyms straight. I would recommend writing out the insecticide treatments instead. It would really make the manuscript more accessible. However, if you feel that the acronyms are necessary, please consider simplifying them. Can you simplify Py-PBO to just PBO? Having such long acronyms makes deciphering the treatments tiring.

**Response**
We have change consistently Py-PBO LLIN to PBO-LLIN

iii. Ln 70: Please make sure to define IRS, Indoor residual spray, for the reader. I would also elaborate on this approach too. Why is it used? How does it compare to netting? Does combining approaches and insecticide classes affect resistance and resistance management? I would also reiterate here that the pyrethroid used in the netting was permethrin.

**Response**
We define IRS as Indoor residual spraying. However, IRS is a common intervention in malaria vector control, and we would prefer not to expand as It is out of scope of the paper. Readers could read the RCT trial results paper cited above if they want to learn more about IRS and the relative success of IRS vs bednets.

iv. Lns 91-94: At this stage it would be helpful describe in detail what you hypotheses were. What did you expect to learn from the deployment of the different treatments to the 5 villages in Muleba? Summarize in a few sentences what you did and why.

**Response**
In contrast to the Lancet trial paper where this is described, the present work was not about deploying different treatments in the five villages but it aimed at characterizing molecular and metabolic resistance mechanisms present in intensely pyrethroid resistant populations of *An. gambiae* and *An. funestus*, and sought to describe the phenotypic and genetic insecticide resistance profiles of malaria vectors in Muleba.

We have added the following at the end of the Introduction to make it clearer: 'We hypothesize that the frequency of pyrethroid resistance in Muleba populations of *An. gambiae s.s* and *An. funestus* is high, accompanied by over expression of key candidate P450s genes.'

v. Ln 96: Why is this first section of the methods in quotes? Please remove. I would also give this section a heading of its own "Consent and ethical clearance" or something to that effect.

**Response:**
Done. The quotes have been removed and the content has been moved to **Materials and methods** section above **Data analysis section**; titled **Consent and ethical clearance**, as shown via tracked changes

vi. Ln 104-117: There is a lot of information missing from this section. Please include 1) the dates that each treatment were deployed, 2) how long they were deployed, 3) whether and when the treatments were reapplied or serviced, 4) the names of the pesticides used, 5) the manufacturer information of those pesticide products, 6) the netting information (mesh gauge, brand), 7) the concentrations and volume applied both to the netting and the indoor sprays.

Response: We have mentioned the different RCT treatment in the introduction when presenting the main trial results, and they are also mentioned in the material and methods. This was requested by a previous reviewer. We have added in the revision the date IRS and LLIN were deployed (February 2015). However as mentioned in responses above (i and iv) we did not aim to present any efficacy data, and specific information on pesticide product characteristics is not relevant for this paper. All additional information related to the main trial can access the publication online.

vii. Figure 1: Can you include the sampling location data on the map? How many sample sites were in each village? Just one or multiple?

**Response:**
Mosquitoes were collected in several houses per village, but we are not able to include sampling location as we did not take coordinates of the houses. All mosquitoes from the same village were pooled together for the testing.

viii. Ln 119: Please reconsider this section organization. I think there this too much here and some of the information belongs in a different section altogether. Please only include sample collection and id info here. Move all the information in the middle paragraph to a new section on lab susceptible strains.

**Response:**
As suggested, we have reorganized and present two sub headings, one with the wild mosquitoes and the second one with the laboratory strain mosquitoes.

ix. Ln 121: What how many samples were collected? For the data to be robust (fig2) there should be multiple collections from multiple sites in each village. Was this done? If so, please describe.

**Response:**
Mosquitoes were collected in different houses and for each test as mentioned in material and methods we have indicated in the methods that 'Approximately 100 mosquitoes (25 per replicate) were used per test.' (cf under Resistance assay with diagnostic concentration line 188). The number of mosquitoes exposed per insecticide and location varied between 89 to 171. This sentence was added in the result section (under Resistance assay with diagnostic concentration).

x. Ln 127 and going forward: Please refrain from referring to these strains by their village names. It makes keeping them straight nearly impossible for the reader. Instead, please refer to them as the susceptible strain for each species.

**Response:**
The paragraph is about the wild mosquitoes collected in different villages and identification and should not be confused with the susceptible strains, a new section for laboratory susceptible strains is now in place. All this information was added to respond to a previous reviewer.

xi. Ln 141: For each of these sections going forward I would consider giving a brief 1-2 sentence description of why these assessments were conducted. This helps the reader understand why you did what you did and better interpret the results later on. Including a clear description of your hypotheses in the introduction will help with this too.

**Response:**
Description given is now given as suggested, as shown in tracked changes

xii. Ln 144-145: Because you refer to the class of insecticides throughout the article, it would be helpful at this stage to include those class names here with each of the insecticides you tested. For instance, you could include in parentheses (pyrethroid) after permethrin, or (carbamate) after bendiocarb.

**Response:**
The names have been included as suggested, as shown in tracked changes

xiii. You also need to include the source of the chemicals you used here. Where did you purchase them? Who was the manufacturer? This is important for replication. What are these concentrations in ug/ml values? This will help these data make sense given the concentration values reported later on in fig. 3.

**Response:**

We have mentioned that all papers were procured from the WHO recommended supplier; the Universiti Sains Malaysia (ln 187). The concentrations of those papers are given in brackets for each insecticide tested. The concentrations of those papers are expressed in percentages not in µg/ml. Expression of concentrations in µg/ml is used in CDC bottle bioassays, a different technique for assessing susceptibility / resistance status of wild mosquitoes.

xiv. I agree with one of the other reviewers too, that including information on why these insecticides were selected for analysis is important. Right now that information is absent. Is bendiocarb at risk for cross resistance? Are lambda-cyhalothrin and bendiocarb used in mosquito management and if so how? Water treatments? Fogging? Netting?

**Response:**

We have added a sentence that *"Those insecticides are currently used for LLIN and IRS treatment."*

xv. Ln 152: Why were PBO and permethrin administer separately rather than concomitantly? This method is not explained.

**Response:**

Synergy testing was based on the standard CDC Bottle Bioassay Technique (CDC, 2010) that requires pre-exposure of mosquitoes to PBO followed by insecticide exposure. This reference is reported.

xvi. Ln 166: Please be sure to fully explain what the goals of each genetic analysis were. In other words, why do we care about these mutations?
Also please include the collection dates and location for the insects used in these analyses.

**Response:**

We added to/modified the sentence at the beginning of the genotyping section 'We genotyped kdr and GSTe2 mutations in F1 progeny from field-collected An. gambiae s.s. and An. funestus, respectively; each of these mutations have proven causative links with pyrethroid resistance (Riveron et al. 2014; Donnelly et al. 2016)
Collection dates and locations have been added and we have proposed to add a figure in supplementary file for testing timeline (see below)

"Synergist assays with piperonyl butoxide (PBO) were undertaken in 2017 to identify the potential role of elevated mixed-function oxidases in resistance in *An. gambiae s.l.* (Kakoma) and *An. funestus* (Kabirizi)."

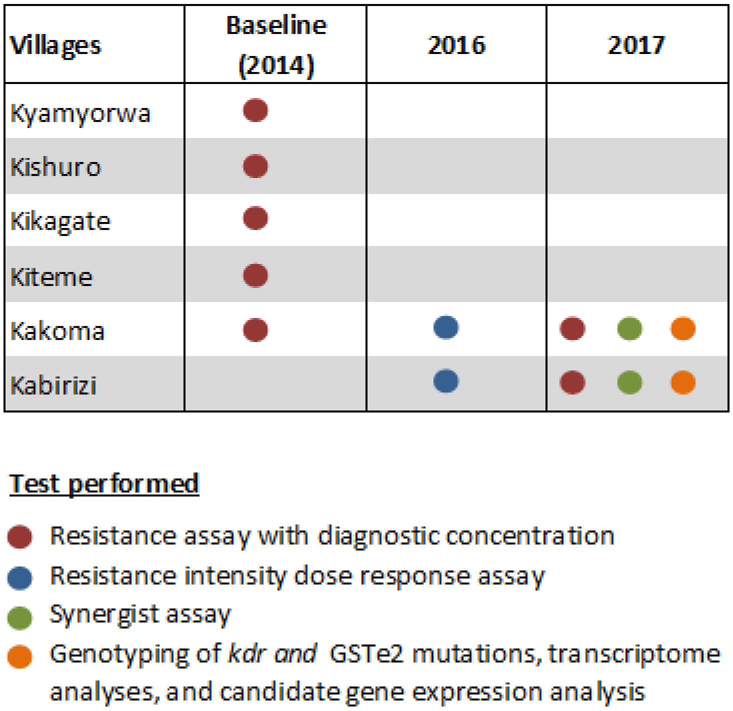


As a result, we have added a figure **(S1 Fig.) for Testing timeline.**
In this case, the previous **S1 Fig.** for **Interwoven microarray experimental loop design** has become **S2 Fig.**

xvii. Ln 182: Same as the last comment. Please describe why you conducted this transcriptome analysis, what you attempted to learn, and why that is important. Also please include collection dates and location.

**Response:**
We have added the following sentence
"Transcriptome analyses were carried out to identify genes that were putatively involved in observed insecticide resistance in Muleba populations of An. gambiae s.s. and An. funestus. Both An. gambiae s.s. and An. funestus were collected in May-June 2017 from Kakoma and Kabirizi villages respectively. "

xviii. Ln 246: Is there no trapping data you can include here? How many flies were recovered from each village during each sample? How did you evaluate the efficacy of each treatment?

**Response:**

We did not aim to evaluate the efficacy of each intervention as part of the work presented here. Hundreds of mosquitoes that were collected from each village were tested in WHO susceptibility tests

xiii. Ln 251: Please refer to the treatments instead of or alongside the village names.

**Response:**

We prefer to keep referring to the village where mosquitoes were collected. Information on type of intervention for each villages was indicated to give more background information and requested by one of the previous reviewers.

xiv. Ln 253: Please report how many flies were collected at each location, if possible.

**Response**: We did not record the total mosquitoes by each location; we just indicated the number of mosquitoes tested as the sole purpose of the mosquito collections was for resistance testing.

xv. Lns 257-265: These is a lot of information here. I could consider making a table instead and show the Lc50 values of each species at each location for each year.

**Response:**

The narration is on phenotypic resistance results (percentage mortalities following exposure of mosquitoes to diagnostic doses using the standard WHO guideline for susceptibility testing). The $LC_{50}$ values have been reported in table 1 following regression analysis of the CDC bottle bioassays data.

xvi. Ln 277: What was the diagnostic dose? Providing this information is important for comparison.

**Response:**

The diagnostic dose has been included, as shown in tracked changes

xvii. Ln 289: Please consider adding an additional footnote to table 1. Please include how RR is calculated. I would also consider adding information to the methods and materials describing the significance of different RR values. The WHO describes that values less than 5 indicated susceptibility while values greater than 5 indicate resistance in Aedes mosquitoes. Is this the same for Anopheles?

**Response:**

We have added the following sentence in the analysis section "A resistance ratio of 2 indicates potential resistance or suggestive of probable resistance while resistance ratio greater than 2

indicates resistance". Furthermore, according WHO a concentration of 10 times the diagnostic dose would be interpreted as indicative of high resistance intensity [26]. This is mentioned in the discussion.

xviii. Fig. 2. Please add statistical analysis between groups. Logistic regression and posthoc analysis among sites for each pesticide would help determine if these differences in mortality were in fact different.
I would also add the species label to the y-axis for clarity, and change the village names to treatment labels

**Response:**

- We prefer to keep the descriptive analysis as we did. We do not have enough data to be able to have meaningful comparisons. Furthermore fig 2 corresponds to data collected at baseline.
- The y-axis is for 24hrs percentage mortality
- As in our response above, we prefer to keep the villages names, we do not mean to associate the result to a treatment as this was not the purpose of the paper.

xix. Fig. 3. I would again, go back and use logistic regression to compare survival and report he LC50 values here on the figure. For good measure, you could also denote the diagnostic dose here on the figure for each species.

**Response:**

- The LC50 values for each species have been reported in table 1
- The diagnostic dose for CDC Bottle Bioassay is the same for all *Anopheles* species and has now been stated

xx. Fig 4. In figure captions, please refer to treatment at the Kabirizi village. Also include what year those samples were collected.
Can you conduct a comparison of the CT values for Kabarizi vs the lab susceptible population (FANG)? A t-test for each gene may suffice. Otherwise, we don't know if these differences are statistically significant or not.

**Response:**

- The treatment and year in which mosquitoes were collected at Kabirizi are stated in methods section (we have also added a figure to clarify)
- Yes, a comparison of the CT values for *An. funestus* that were collected Kabirizi vs the lab susceptible population (FANG) was done using t-test as stated in Data analysis section

xxi. Fig. 5. What year were these samples collected? Refer to the lab susceptible populations rather than village names (Kisumu or Ngousso). Again, run a stats comparison for the village vs lab susceptible. Also, the DPI resolution of this figures is a little low, making the figure labels difficult to read, but that could just be on my end.

**Response:**

- The year in which mosquitoes were collected at Kakoma are stated in methods section
- The village name (Kakoma) represents the wild/field mosquitoes (*An. gambiae s.s*) that were collected from that village. The CT values for *An. gambiae s.s* from Kakoma were compared with the average values of the susceptible strains (Kisumu and Ngousso) using using t-tests

xxii. Ln 358: This sentence appears to have a typo. Change to " The addition of PBO in the synergist assay…" ?

**Response:**

Corrected, see the tracked changes

xxiii. Ln 380: The authors state that the mechanism of resistance appears the same for the pyrethroids and carbamate because bendiocarb resistance was present in An. funestus. However, this may not be the case and the experiments reported here to do not evaluate this hypothesis. Be careful not to overextend on your claims.

**Response:**

Noted, thank you

xxiv. Ln 389: Where are these data reported? Where does this 90% values come from?

**Response:**

The frequency of pyrethroid resistance (90%) is reported in figure 2 of the manuscript

***We thank the reviewers for their helpful comments***
