# Supplementary material for: Expression of pyrethroid metabolizing P450 enzymes characterizes highly resistant Anopheles vector species targeted by successful deployment of PBO-treated bednets in Tanzania
Source: PLoS One. 2022 Jan 24;17(1):e0249440. doi: 10.1371/journal.pone.0249440 (PMC8786186; doi:10.1371/journal.pone.0249440)
Supplement: Attachment — Submitted filename: Rebuttal letter_Dec. 2021.pdf [file pone.0249440.s013.pdf]

Dear Editor,

We have revised our paper in line with the reviewers' and editorial comments. We hope the changes and responses meet the expectation of Plos One and the manuscript is now suitable for publication.

Yours sincerely,

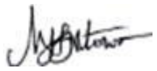

Dr. Johnson Matowo (PhD)

Response to academic editor and reviewer(s)

a) Response to academic editor

- No changes have been made to the reference list
- No retracted papers have been cited
- The last remaining issues with special attention to statistics and enough information on other variables already published have been addressed

Additional comments to academic editor

There was an accidental omission of two supplementary tables that show the full microarray results for *An. funestus* and *An. gambiae s.s.* These have been added as S3 Table and S6 Table respectively. Following this, re-numbering of S Tables has been done accordingly (where they are listed at the end of the manuscript). Furthermore, all S Tables have been referred to correctly in the text.

b) Response to reviewer(s)

i. All comments that were raised in a previous round of review by Reviewer #1 & #2 had been addressed

ii. The following are responses to Reviewer #03

Reviewer #3:

- Line 32 (in the abstract) has been rephrased to exclude the word efficacy, targeting resistance mechanisms
- The comma after the word study (Line 103) has been removed
- The quotation marks have been removed (Lns 112-118)

- Line 124-126 (Not Ln 110-124 as indicated by reviewer): Few sentences have been added to describe the study, the names of the pesticides, suppliers and their concentrations/ application dosages. In addition, the readers have been referred to Protopopoff 2018 i.e. [16].
- Information on analysis of differences in CT values between groups using t-tests has been included in Data analysis section of the manuscript
- The t values and p-values describing significant differences among treatments using t-tests have been included in S5 Table for *An. funestus* and S8 Table for *An. gambiae s.s*

We thank the reviewer #03 for his/her helpful comments!!!
